# α-Linolenic acid but not linolenic acid protects against hypertension: critical role of SIRT3 and autophagic flux

**DOI:** 10.1038/s41419-020-2277-7

**Published:** 2020-02-03

**Authors:** Guohua Li, Xinpei Wang, Hongyan Yang, Pengfei Zhang, Fangqin Wu, Yunchu Li, Yingjie Zhou, Xing Zhang, Heng Ma, Wei Zhang, Jia Li

**Affiliations:** 1 School of Aerospace Medicine, Fourth Military Medical University, Xi’an, 710032 China; 2 Department of Physiology and Pathophysiology, School of Basic Science, Fourth Military Medical University, Xi’an, 710032 China; 3 Department of Cardiology, Tangdu Hospital, Fourth Military Medical University, Xi’an, 710032 China

**Keywords:** Fatty acids, Hypertension

## Abstract

Although dietary α-linolenic acid (ALA) or linolenic acid (LA) intake was reported to be epidemiologically associated with a lower prevalence of hypertension, recent clinical trials have yielded conflicting results. Comparable experimental evidence for the roles of these two different fatty acids is still lacking and the underlying mechanisms need to be further elucidated. Our data showed that ALA but not LA supplementation alleviated systolic blood pressure elevation and improved ACh-induced, endothelium-dependent vasodilation in both spontaneously hypertensive rats (SHRs) and AngII-induced hypertensive mice. In addition, SHRs displayed reduced vascular Sirtuin 3 (SIRT3) expression, subsequent superoxide dismutase 2 (SOD2) hyperacetylation and mitochondrial ROS overproduction, all of which were ameliorated by ALA but not LA supplementation. In primary cultured endothelial cells, ALA treatment directly inhibited SIRT3 reduction, SOD2 hyperacetylation, mitochondrial ROS overproduction and alleviated autophagic flux impairment induced by AngII plus TNFα treatment. However, these beneficial effects of ALA were completely blocked by silencing SIRT3. Restoration of autophagic flux by rapamycin also inhibited mitochondrial ROS overproduction in endothelial cells exposed to AngII plus TNFα. More interestingly, SIRT3 KO mice developed severe hypertension in response to a low dose of AngII infusion, while ALA supplementation lost its anti-hypertensive and endothelium-protective effects on these mice. Our findings suggest that ALA but not LA supplementation improves endothelial dysfunction and diminishes experimental hypertension by rescuing SIRT3 impairment to restore autophagic flux and mitochondrial redox balance in endothelial cells.

## Abstract (web summary)

Schematic diagram of the mechanisms whereby α-linolenic acid protects against endothelial dysfunction and hypertension.

## Graphical abstract

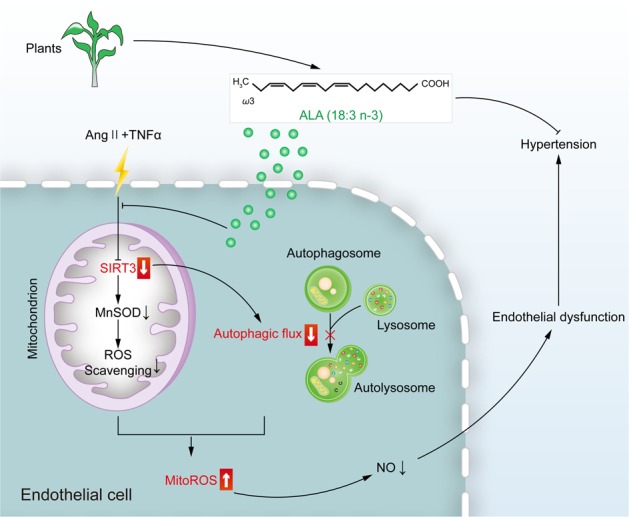

## Introduction

As the leading risk factor for cardiovascular disease, hypertension has become the most prevalent chronic disease, affecting more than 30% of adults aged ≥25 years worldwide^1^. Considering its high prevalence and consequent global disease burden, preventive strategies from a public health perspective are urgently needed, and nutritional interventions have been advanced to fight hypertension epidemic^2^.

As a plant-derived omega-3 polyunsaturated fatty acid (n-3 PUFA), α-linolenic acid (ALA) is abundant in nuts, leafy vegetables and plant seed oils, such as rapeseed, soyabean and flaxseed oils^3,4^. Dietary ALA intake was reported to be epidemiologically associated with a lower prevalence of hypertension, and ALA has been indicated as a promising alternative addition to available lifestyle medications for the prevention of cardiovascular diseases^5,6^. In contrast to the recognized benefits of n-3 PUFAs, diets enriched with omega-6 (n-6) PUFAs have traditionally been viewed as detrimental primarily because they are precursors for pro-inflammatory eicosanoids^7^. However, a recent epidemiological study reported that higher plasma levels of n-6 PUFA linolenic acid (LA) were significantly associated with a lower prevalence of hypertension^8^. Therefore, comparative data and strong experimental evidence are urged to draw a conclusion about the effects of both dietary n-3 and n-6 PUFAs on hypertension.

The Sirtuins are a family of NAD^+^-dependent deacetylases and ADP-ribosyltransferases, among which Sirtuin 3–5 (SIRT3-5) are located in the mitochondria. Mitochondrial Sirtuins regulate numerous aspects of mitochondrial biology, including metabolic homeostasis, redox balance and mitochondrial dynamics^9^. The associations between mitochondrial Sirtuins impairment and cardiovascular disease have been well established by many preclinical studies. In particular, SIRT3 impairment and the resultant SOD2 hyperacetylation have been demonstrated to induce vascular oxidative stress and contribute to the development of hypertension^10^. SIRT3 has also been revealed to regulate the autophagy-lysosome pathway and autophagic flux is implicated in the management of nitric oxide (NO) bioavailability and endothelial function^11,12^. Although the anti-oxidative property of ALA has been reported previously, whether SIRT3 plays an important role in the benefits of ALA remain unknown.

Given the increasing evidence suggesting ALA or LA as a nutritional supplement with cardiovascular-protecting potential, the present study was designed to comparatively investigate the effects of ALA and LA supplementation against hypertension and the underlying molecular mechanisms.

## Materials and methods

### Chemicals and reagents

MitoSOX Red (Ex/Em: 510/580 nm; M36008) and MitoTracker Green (Ex/Em: 490/516 nm; M7514) probes were supplied by Invitrogen (Carlsbad, CA, USA). SIRT3 (ab118334), acetyl-K68-SOD2 (ab137037), SOD2 (ab68155), p62 (207305), LC3B (ab63817) and β-actin (ab6276) antibodies were obtained from Abcam (San Francisco, CA, USA). All other chemicals and reagents were obtained from Sigma (St Louis, MO, USA).

### Animal experiments

All procedures involving animals were performed according to the National Institutes of Health Guidelines for the Use of Laboratory Animals, and were approved by The Fourth Military Medical University Committee on Animal Care. Four-week-old male spontaneously hypertensive rats (SHRs) and age-matched normotensive Wistar-Kyoto (WKY) control rats were purchased from Vital River Laboratory (Beijing, China). The control diet (no.AIN-93G; 20% protein, 64% carbohydrate and 16% fat), LA-supplemented diet (no.AIN-93G + 10% safflower oil; 20% protein, 54% carbohydrate, and 26% fat) and ALA-supplemented diet (no.AIN-93G + 10% flaxseed oil; 20% protein, 54% carbohydrate and 26% fat;), as reported previously^13,14^, were obtained from Research Diets (New Brunswick, NJ). The animals were randomly divided into four groups with different diets for 8 weeks using a completely randomized design: (1) WKY: the normotensive WKYs fed with the control diet; (2) SHR: the SHRs fed with the control diet; (3) SHR + LA: the SHRs fed with LA-supplemented diet; (4) SHR + ALA: the SHRs fed with ALA-supplemented diet. Food consumption of the SHRs fed with the control diet was firstly recorded by weight, and these data were used to pairfeed animals on the LA-supplemented diet and ALA-supplemented diet to maintain caloric intake. Diet fatty acid composition was confirmed by gas chromatography.

For angiotensin (Ang) II-induced hypertensive model, male eight-week-old C57BL/6J mice were fed with the ALA-supplemented diet or control diet for 4 weeks, and then randomized to receive a low-suppressor dose of AngII (0.4 mg/kg/d, 2 weeks) or vehicle via an osmotic minipump (1002, Alzet, USA) as described previously^10^. SIRT3 knock-out (SIRT3KO) and wild-type (WT) mice were obtained from Jackson Laboratory (Bar Harbor, Maine, USA), and hypertension was also induced by AngII infusion (0.4 mg/kg/d, 2 weeks).

### Measurement of blood pressure

Systolic blood pressure was measured in conscious animals by tail-cuff system (BP-98A, Softron) to monitor the progression of hypertension. At the end of the experiments, systolic blood pressure was assessed by inserting a heparinized saline-filled catheter into the left carotid artery after an initial 15-min equilibration period in anaesthetized animals. Blood pressure was measured between 9:00 a.m. and 11:00 a.m.

### Biochemical parameter measurement

Blood samples were obtained from rats after overnight fasting. Fasting blood glucose and insulin levels were respectively measured by a blood glucose meter (Lifescan) and an ELISA test kit (no. 90010, Crystal Chem). Plasma NO was evaluated by the NO fluorometric assay (no. K252-200, BioVision) according to the manufacturer’s instructions.

For fatty acid analysis, 250 μL aliquots of serum from different group animals were analyzed and quantified by GC-MS (QP 2010 ultra) as described previously^15^.

### Vascular function assessment

Mice were sacrificed and the descending aorta was carefully excised and placed in ice-cold physiological saline solution (PSS) as described previously^16^. Experiments were performed in a horizontal wire myograph (DMT, Aarhus, Denmark) containing PSS. The contractile force was recorded using a PowerLab Chart v7.2.1 program (model 610 M, Danish Myo Technology, Denmark). After a 60-min equilibration period, one dose of physiological saline solution containing 60 mM KCl (KPSS) was administered to verify vessel viability. Aortic rings were precontracted with phenylephrine (PE, 10^−5^ M, Sigma, USA). Endothelium-dependent vasorelaxation evoked by acetylcholine (ACh, 10^−9^ to 10^−5^ M, Sigma, USA) and endothelium-independent vasorelaxation evoked by cumulative sodium nitroprusside (SNP, 10^−10^ to 10^−5^ M, Sigma, USA) were expressed as percent contraction determined by the percentage of inhibition to the precontracted tension.

In another experiment, ACh-induced, endothelium-dependent vasorelaxation and SNP-induced, endothelium-independent vasorelaxation in 2 mm mesenteric artery rings isolated from WKYs and SHRs fed with control diet, LA-supplemented diet or ALA-supplemented diet were measured after precontracted with PE (10^−5^ M, Sigma, USA).

### Cell culture

Human aortic endothelial cells (HAEC) were purchased from Lonza (Chicago, IL) and cultured in EGM-2 medium supplemented with 2% FBS without antibiotics. The cell line has been routinely checked for mycoplasma contamination with the MycoProbe detection kit (no. CUL001B, R&D system). Only cells negative for mycoplasma contamination were used.

### Assessment of ROS production

Stock solutions of MitoSOX (4 mM; M36008, Invitrogen, USA) in DMSO were prepared and were diluted in Kreb-Hepes buffer to a final concentration of 2 μM MitoSOX. Production of mitochondrial O_2_^•−^ was measured as accumulation of mitochondrial 2-hydroxyethidium in MitoSOX supplemented samples as described previously^10^. Three 2-mm aortic rings from each animal are incubated in 2 μM MitoSOX for 30 min at 37 °C. Next, buffer was aspirated and the tissue is then homogenized in 300 μL of methanol, and 50 μL of homogenate are used for protein determination. The remainder of the sample is passed through a 0.22-μm syringe filter and then used for HPLC analysis. MitoSOX and its oxidation products, 2-hydroxyethidium and ethidium, were separated using a C-18 reverse-phase column (Nucleosil 250 to 4.5 mm) and a mobile phase containing 0.1% trifluoroacetic acid and an acetonitrile gradient (from 37 to 47%) at a flow rate of 0.5 ml/min. Ethidium and 2-hydroxyethidium were detected with a fluorescence detector using an emission wavelength of 580 nm and an excitation of 480 nm. The mitochondrial 2-hydroxyethidium peak reflects the amount of mitochondrial O_2_^•−^ formed in the tissue during the incubation and is expressed per milligram of protein. H_2_O_2_ was measured by Amplex™ Red Hydrogen Peroxide/Peroxidase Assay Kit (Thermo Fisher A22188) according to the manufacturer’s instructions.

Immunofluorescent staining of mitochondrial ROS was also performed on aortic frozen sections from WKYs and SHRs fed with different diets. Aortic frozen sections were firstly permeabilized with 0.4% Triton X-100 in PBS for 10 min, and then blocked with 10% goat serum in PBST containing 0.1% Tween-20 for 1 h at room temperature. Aortas were incubated with rabbit anti-CD31 (NB100-2284, Novus, USA) primary antibody overnight at 4 °C, followed by incubation of MitoSOX at the concentration of 10 µM for 20 min. Images were acquired using a Zeiss (LSM 800) confocal microscope.

In vitro, production of mitochondrial O_2_^•−^ was visualized in intact cultured HAECs using the fluorescent probe MitoSOX. HAECs were incubated with 10 μM MitoSOX in Kreb-Hepes buffer for 20 min at 37 °C in a CO_2_ incubator. The mitochondrial subcellular location of MitoSOX was confirmed by co-labeling with 50 nM MitoTracker Green FM (Ex/Em: 490/516 nm, M7514, Invitrogen, USA).

### mCherry-GFP-LC3 assay

Autophagy was visualized in HAECs by transfection of the lentivirus expressing tandem mCherry-GFP-LC3 (tf-LC3, Hanbio Biotechnology, China). GFP fluorescence is quenched in the acidic pH of the lysosomal compartment, thus limiting the application of GFP-LC3 to the identification of autophagosomes. However, mCherry continues to fluoresce, and mCherry-GFP can be applied to the identification of both autophagosomes and autolysosomes. Quantification of autophagosomes can be achieved by using tf-LC3 and determining the number of red dots that overlay green dots and appear yellow in merged images. The red dots which do not overlay green dots and appear red in merged images indicate autolysosome formation. After infection of tf-LC3 lentivirus for 24 h, endothelial cells were subjected to the corresponding treatment and then imaged for GFP and RFP by using a Zeiss (LSM 800) confocal microscope.

### siRNA transfection

The siRNA-mediated SIRT3 knockdown was performed by transfecting synthetic siRNA duplexes (Genepharm, Shanghai, China) at a concentration of 20 nM with Lipofectamine RNAiMAX regent (Invitrogen, Carlsbad, CA, USA) according to the protocols provided by the manufacturer. The siRNA sequences are as follows: human SIRT3, 1. sense 5′-CCAGCAUGAAAUACAUUUATT-3′, anti-sense 5′-UAAAUGUAUUUCAUGCUGGTT-3′. 2. sense 5′-CCAGUGGCAUUCCAGACUUTT-3′, anti-sense 5′-AAGUCUGGAAUGCCACUGGTT-3′. Scarmbled siRNA, sense 5′-UUCUCCGAACGUGUCACGUTT-3′, anti-sense 5′-ACGUGACACGUUCGGAGAATT-3′. The transfected cells were cultured for 48 h and used for further experiments.

### Statistics

Data were presented as mean ± standard error of the mean (n noted in specific figure legends). The sample size was determined based on previous studies with similar experimental design and on the known variability of the assay. Analysis of the immunofluorescence-related data was performed in a blinded way. Normality of data distribution was assessed by Shapiro-Wilk normality test. An unpaired, two-tailed Student *t* test (two groups) or ANOVA (three or more groups), followed by Bonferroni’s correction if needed, were performed to determine statistical significance between different treatment groups. The data of blood pressure over the time course were statistically analyzed by repeated measures analysis of variance. *P* levels of <0.05 were considered significant.

## Results

### ALA but not LA supplementation exerted anti-hypertensive and endothelium-beneficial effects in experimental hypertensive animals

**Fig. 1:**
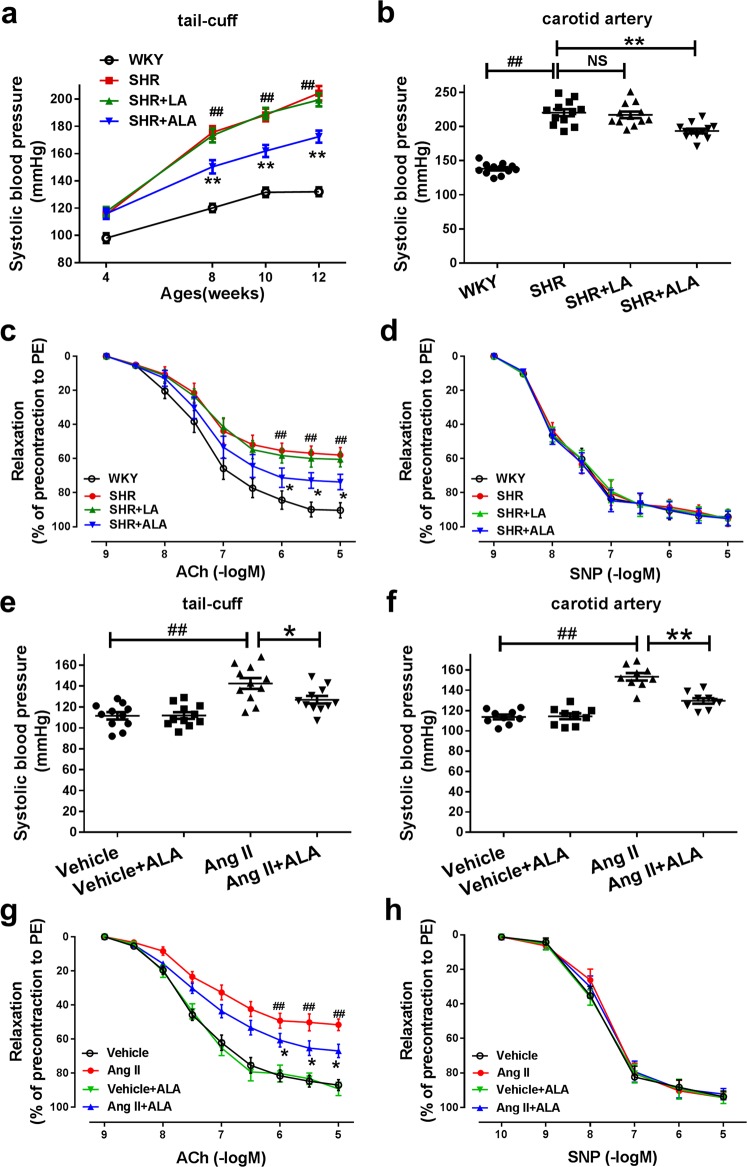
ALA but not LA supplementation attenuated endothelial dysfunction and blood pressure elevation in experimental hypertensive animals. **a** Systolic blood pressure measurement by tail-cuff methods in WKYs and SHRs fed with different diets for 8 weeks. **b** Direct catheter measurement of systolic blood pressure in WKYs and SHRs after 8 weeks of different diets. *n* = 12 animals per group. **c, d** Concentration-response curves for acetylcholine (ACh) and sodium nitroprusside (SNP) in mesenteric arteries from WKYs and SHRs fed with different diets. *n* = 9 animals per group. Four-week-old male SHRs were fed with the control diet, LA-supplemented diet or ALA-supplemented diet for eight weeks respectively. Data are expressed as means ± SEM; ^##^*P* < 0.01 vs. WKY. ^*^P < 0.05, ^**^P < 0.01 vs. SHR. **e** Systolic blood pressure measurement by tail-cuff methods in AngII-induced hypertensive mice and vehicle-infused normotensive mice fed with ALA or control diet. n = 11 animals per group. **f** Direct catheter measurement of systolic blood pressure in different groups at the end of the administration. *n* = 9 animals per group. **g, h** Concentration-response curves for ACh (*n* = 9 animals/group) and SNP (*n* = 6 animals/group) in aortas from AngII-induced hypertensive mice and normotensive mice fed with ALA or control diet. Eight-week-old mice were fed with control or ALA-supplemented diets for 4 weeks, and then randomized to receive AngII (0.4 mg/kg/d) or vehicle for 2 weeks via an osmotic minipump. Data are expressed as means ± SEM; ^##^*P* < 0.01 vs. Vehicle. ^*^P < 0.05, ^**^P < 0.01 vs. AngII.

In order to clarify the potential beneficial effects of diets enriched with n-3 or n-6 PUFAs on SHRs, four-week-old male SHRs were respectively fed with the control diet, LA-supplemented diet or ALA-supplemented diet for 8 weeks. There was no significant difference in body weight among various groups of SHRs (Supplementary Fig. S1a). Serum fatty acids were analyzed among all these different group animals. The SHRs supplemented with ALA had significantly decreased serum levels of arachidonic acid (AA) and significantly increased serum levels of n-3 fatty acids (ALA, EPA and DHA) out of the total fatty acids. In contrast, the SHRs supplemented with LA had significantly increased serum levels of n-6 fatty acids (LA and AA), thus validating the effectiveness of our dietary intervention (Supplementary Fig. S1b). As shown in Fig. 1a, there was no difference among various groups of SHRs in baseline systolic blood pressure (SBP). After 8 weeks of diet treatment, ALA supplementation significantly reduced SBP measured by either tail-cuff or carotid artery catheterization method in SHRs, and in contrast, LA supplementation showed no obvious effects (Fig. 1a, b). In addition, SHRs had significantly higher fasting serum insulin level than age-matched WKY rats (Supplementary Fig. S1c), although the fasting serum glucose concentration was normal and comparable to that of WKY rats (Supplementary Fig. S1d). Eight-week ALA but not LA supplementation significantly reduced fasting insulin level (Supplementary Fig. S1c), but did not alter fasting glucose concentration in SHRs compared with the control diet (Supplementary Fig. S1d).

Although SHR is by far the most widely used hypertensive animal model, it reflects only a rare subtype of human hypertension^17^. There was a question as to whether the anti-hypertensive effect of ALA is limited to the SHR model. Therefore, eight-week-old mice were fed ALA or control diet for four weeks, and then randomized to receive AngII (0.4 mg/kg/d, 2 weeks) or vehicle via an osmotic minipump. Infusion of wild-type C57Bl/6J mice with a low dose of AngII led to a mild but significant increase in SBP to approximately 140 mmHg (Fig. 1e, f). ALA supplementation reduced SBP in AngII-infused mice, but did not affect blood pressure in vehicle-infused mice, as measured by either tail-cuff or carotid artery catheterization method (Fig. 1e, f).

Of note, ALA supplementation not only reduced blood pressure but also improved endothelial function as evidenced by measurements of endothelium-dependent vasorelaxation evoked by ACh (Fig. 1c, g) in SHRs and AngII-induced hypertensive mice. In contrast, LA treatment did not significantly influence endothelial dysfunction in SHRs, with no obvious alterations of ACh-induced, endothelium-dependent mesenteric artery relaxation (Fig. 1c). In addition, neither ALA nor LA supplementation affected the endothelium-independent vasodilatory responses to SNP (Fig. 1d, h) in SHRs or AngII-induced hypertensive mice. Furthermore, ALA supplementation did not influence ACh-induced, endothelium-dependent vasodilatation in normotensive mice (Fig. 1g). These data have demonstrated that ALA but not LA supplementation prevents blood pressure elevation and improves endothelial dysfunction in SHRs and AngII-induced hypertensive mice.

### ALA but not LA supplementation prevented SIRT3 reduction and SOD2 hyperacetylation and alleviated mitochondrial ROS overproduction in the vasculature of SHRs

**Fig. 2:**
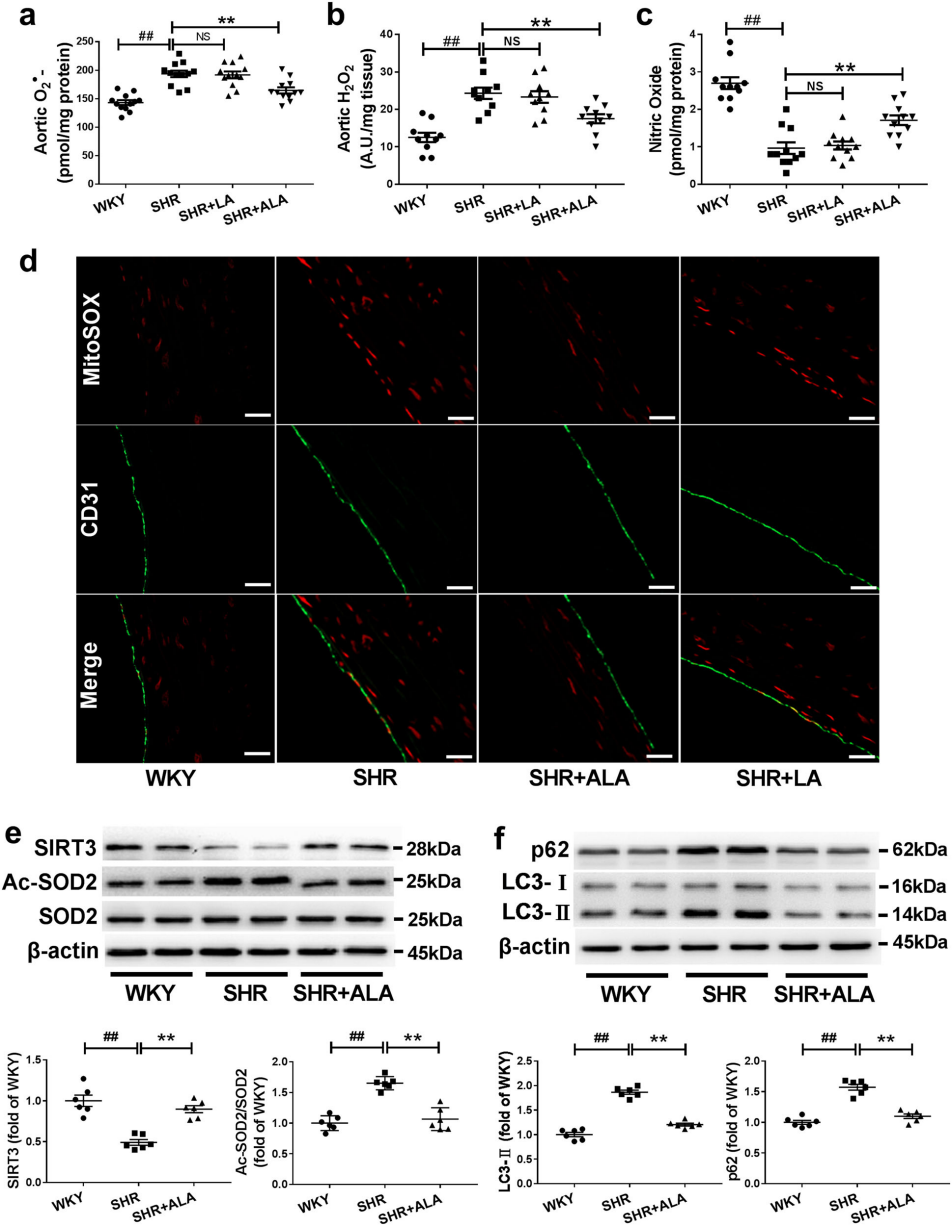
ALA but not LA supplementation prevented SIRT3 reduction and SOD2 hyperacetylation and alleviated mitochondrial ROS overproduction in the vasculature of SHRs. Mitochondrial O_2_^•−^ by MitoSOX/HPLC (*n* = 12 animals/group, **a** H_2_O_2_ levels by Amplex red (*n* = 10 animals/group, **b** and nitric oxide by fluorometric assay (*n* = 11 animals/group, **c** were detected respectively in aortas isolated from SHRs and WKYs fed with different diets. Data are expressed as means ± SEM; ^##^*P* < 0.01 vs. WKY. ^**^P < 0.01 vs. SHR. **d** Representative immunofluorescent staining of mitochondrial ROS (Red) by MitoSOX in aortas from SHRs and WKYs fed with ALA-supplemented, LA-supplemented or control diets, in which endothelium was shown by CD31 staining (Green). Scale bars, 20 µm. **e** Measurement of SIRT3, SOD2 acetylation (Ac-SOD2) and total SOD2 expression by western blot in aortas isolated from SHRs and WKYs fed with ALA-supplemented or control diets. **f** Western blot analysis of aortic p62 and LC3-II expression in SHRs and WKYs fed with ALA-supplemented or control diets. Data are expressed as means ± SEM; *n* = 6 rats per group. ^##^*P* < 0.01 vs. WKY. ^**^P < 0.01 vs. SHR.

Previous data from other labs as well as ours have shown that SIRT3 deficiency and resultant SOD2 hyperacetylation and mitochondrial ROS overproduction contribute to the development of hypertension and vascular dysfunction in obesity^10,18^. On the other hand, ALA has been reported to have an anti-oxidative property. Therefore, we analyzed SIRT3 expression and SOD2 acetylation (K68) by western blot in aortas isolated from SHRs fed different diets. Mitochondrial O_2_^•−^ and H_2_O_2_ levels were respectively detected by MitoSOX/HPLC and Amplex red. SHRs exerted reduced vascular SIRT3 expression (Fig. 2e), enhanced vascular SOD2 acetylation (Fig. 2e), higher vascular mitochondrial O_2_^•−^ (Fig. 2a) and H_2_O_2_ levels (Fig. 2b) compared with WKYs. In addition, MitoSOX Red staining also verified the increased mitochondrial ROS production in the aortas of SHRs, with stronger red fluorescence staining compared with WKYs (Fig. 2d). Of note, ALA supplementation abrogated these changes while LA supplementation showed no obvious effects (Fig. 2a, b, d, e and Supplementary Fig. S2a–c).

Hypertension is commonly associated with a decrease in nitric oxide (NO) bioactivity, due to oxidative inactivation of this radical gas. Indeed, we found that vascular NO level was significantly decreased in SHRs, which was largely rescued by ALA but not LA supplementation (Fig. 2c). Taken together, these data have indicated that ALA but not LA supplementation prevented vascular SIRT3 reduction and SOD2 hyperacetylation, and consequently inhibited mitochondrial ROS overproduction and restored NO bioactivity in SHRs.

### ALA rescued SIRT3 reduction and SOD2 hyperacetylation, and prevented mitochondrial ROS overproduction in endothelial cells exposed to AngII plus TNFα

**Fig. 3:**
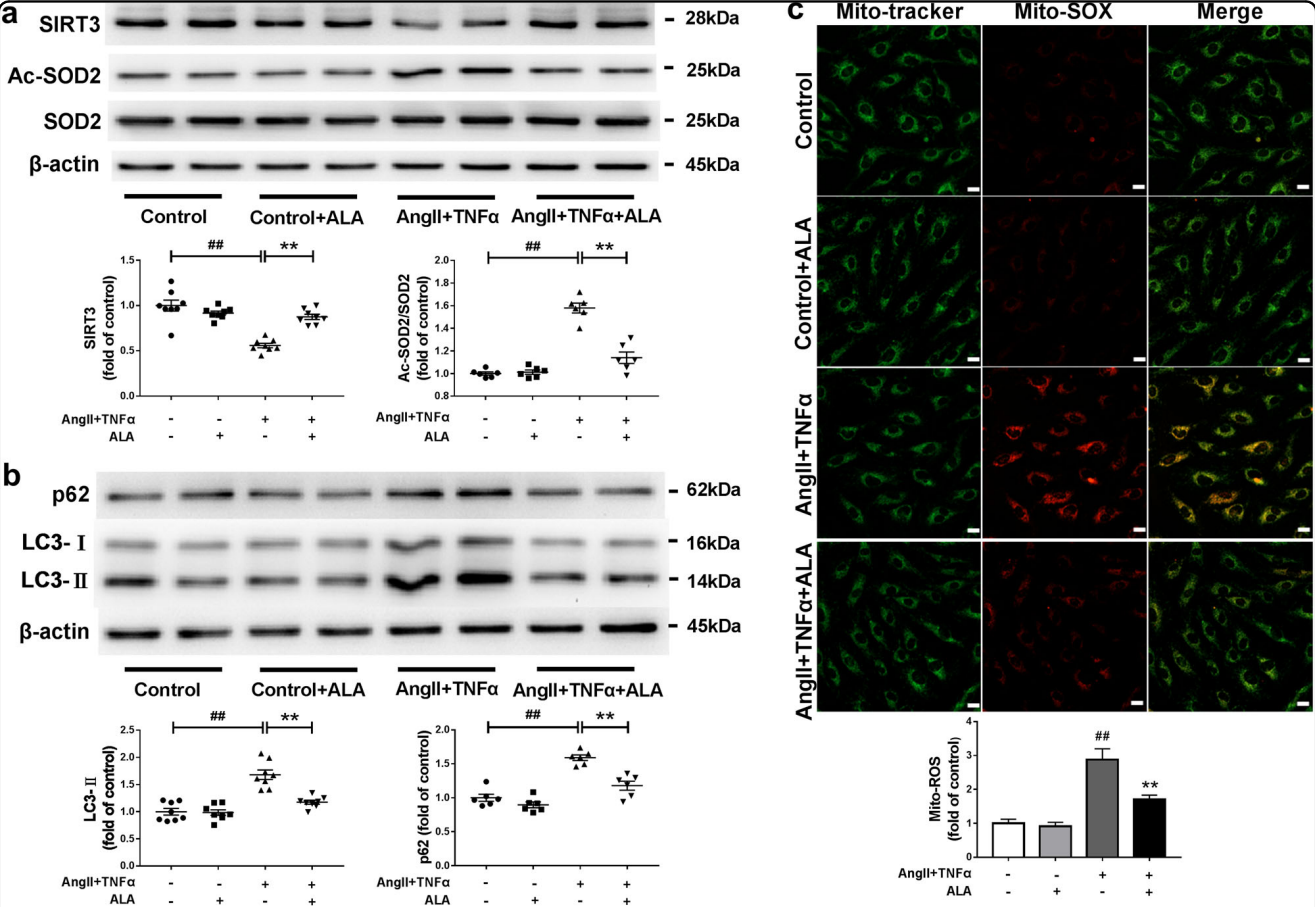
ALA prevented SIRT3 reduction, SOD2 hyperacetylation, autophagy impairment, and mitochondrial ROS overproduction in endothelial cells exposed to AngII plus TNFα. **a** Western blot analysis of SIRT3, Ac-SOD2 and SOD2 expression in endothelial cells. **b** Western blot analysis of p62 and LC3-II expression in endothelial cells. **c** Mitochondria were imaged by MitoTracker Green (100 nM) and mitochondrial ROS production was evaluated by MitoSOX (10 µM, red fluorescence) in endothelial cells. HAECs were exposed to AngII (10 nM) plus TNFα (1 nM) with or without ALA (25 µM) treatment for 24 h. Scale bars, 20 µm. Data are expressed as means ± SEM from 3 independent experiments; ^##^*P* < 0.01 vs. Control. ^**^P < 0.01 vs. AngII + TNFα.

**Fig. 4:**
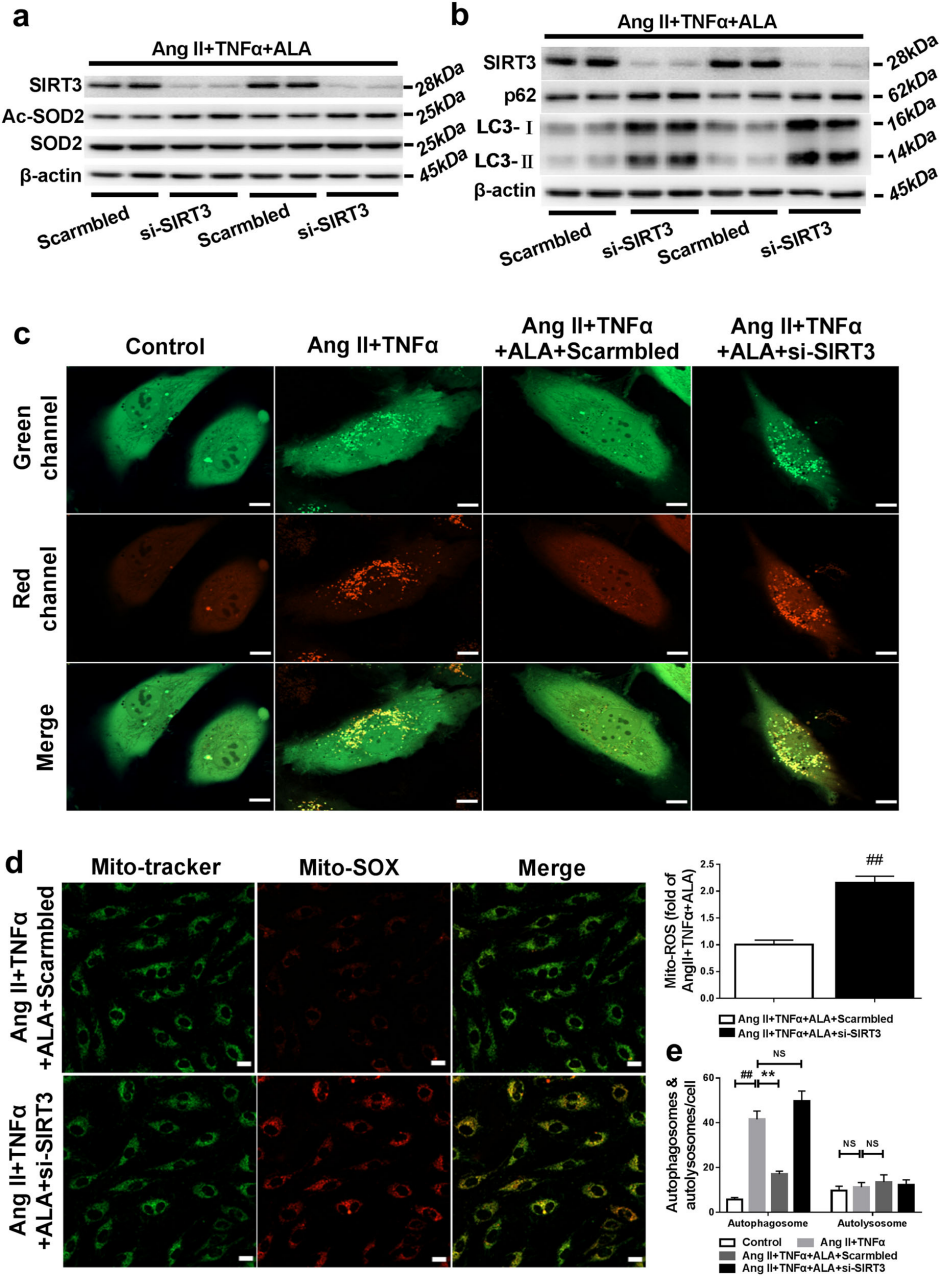
SIRT3 knockdown blunted the beneficial effects of ALA on mitochondrial ROS overproduction and autophagic flux impairment in endothelial cells exposed to AngII plus TNFα. **a** Western blot analysis of SIRT3, Ac-SOD2 and SOD2 expression. **b** Western blot analysis of SIRT3, p62 and LC3-II expression. HAECs transfected with Scambled or SIRT3 siRNA (si-SIRT3) were exposed to AngII (10 nM) plus TNFα (1 nM) with or without ALA (25 µM) administration for 24 h. **c, e** Endothelial cells expressed with mCherry-green fluorescent protein (GFP)-LC3 were imaged by confocal microscope. Yellow dots indicated autophagosomes, while red dots indicated autolysosomes. Quantitative analysis of autophagosomes and autolysosomes in merged imaged per cell are shown. Data are expressed as means ± SEM from 3 independent experiments; ^##^*P* < 0.01 vs. Control. ^**^P < 0.01 vs. AngII + TNFα. Scale bars, 10 µm. **d** Representative immunofluorescent staining of mitochondrial ROS by MitoSOX (10 µM) in HAECs. Scale bars, 20 µm. Data are expressed as means ± SEM from 3 independent experiments; ^##^*P* < 0.01 vs. AngII + TNFα + ALA + Scarmbled.

To determine whether ALA supplementation has a direct benefit on endothelial cells, human aortic endothelial cells (HAECs) were exposed to AngII (10 nM, 24 h) plus TNFα (1 nM, 24 h). ALA dosage (25 µM, 24 h) was chosen in accordance with the plasma levels achieved upon dietary ALA intake with reference to a previous study^19^. Previous studies have indicated that AngII and TNFα are commonly encountered in hypertension^10^. Indeed, AngII plus TNFα administration reduced SIRT3 expression (Fig. 3a), increased SOD2 acetylation (Fig. 3a) and enhanced mitochondrial ROS production (Fig. 3c) in endothelial cells, all of which were prevented by ALA supplementation. In addition, SIRT3 knockdown with specific SIRT3 siRNA blunted the beneficial effects of ALA (Fig. 4a, d). These data have demonstrated that ALA administration directly inhibited SOD2 hyperacetylation and mitochondrial ROS overproduction in endothelial cells exposed to AngII plus TNFα via rescue of SIRT3.

### ALA administration alleviated autophagic flux impairment in endothelial cells exposed to AngII plus TNFα

Disruption of autophagy has been reported to contribute to mitochondrial ROS overproduction^20^. The autophagy markers LC3-II and p62 expression (Fig. 3b), as well as the number of autophagosomes as assessed by GFP-LC3 punctate (Fig. 4c, e), were significantly increased in endothelial cells exposed to AngII plus TNFα, indicating aberrant autophagy. The lysosomal enzyme inhibitors E64d (5 µg/ml) and Pepstatin A (5 µg/ml) were applied to further investigate whether autophagosome clearance was impaired by AngII plus TNFα treatment. Interestingly, blockade of autophagosome clearance by the lysosomal enzyme inhibitors did not further increase LC3-II levels in endothelial cells exposed to AngII plus TNFα (Supplementary Fig. S3a), indicating a defective autophagosome clearance. Furthermore, the lysosomal enzyme inhibitors greatly increased the immunofluorescence staining of mitochondrial ROS by MitoSOX (Supplementary Fig. S3b) in endothelial cells. In comparison, Rapamycin, a potent enhancer of both autophagosome formation and clearance, reduced p62 accumulation and alleviated mitochondrial ROS overproduction in endothelial cells exposed to AngII plus TNFα (Supplementary Fig. S3c and S3d). Taken together, these data have suggested that autophagosome clearance is impaired in endothelial cells exposed to AngII plus TNFα, which might contribute to the overproduction of mitochondrial ROS in these cells.

Notably, ALA administration alleviated autophagic flux impairment as evidenced by decreased LC3-II and p62 accumulation (Fig. 3b) and reduced number of autophagosomes (Fig. 4c, e) in endothelial cells exposed to AngII plus TNFα, all of which were blocked by SIRT3 knockdown with specific SIRT3 siRNA (Fig. 4b, c, e). In addition, the expression of LC3-II and p62 were increased in the aortas from SHRs compared with those from age-matched WKYs (Fig. 2f), both of which were inhibited by ALA supplementation. These data indicated a critical role of SIRT3 in the beneficial effects of ALA on autophagic flux in endothelial cells exposed to AngII plus TNFα.

### Dietary ALA supplementation **e**xerted anti-hypertensive and endothelium-beneficial effects by rescuing SIRT3 impairment

**Fig. 5:**
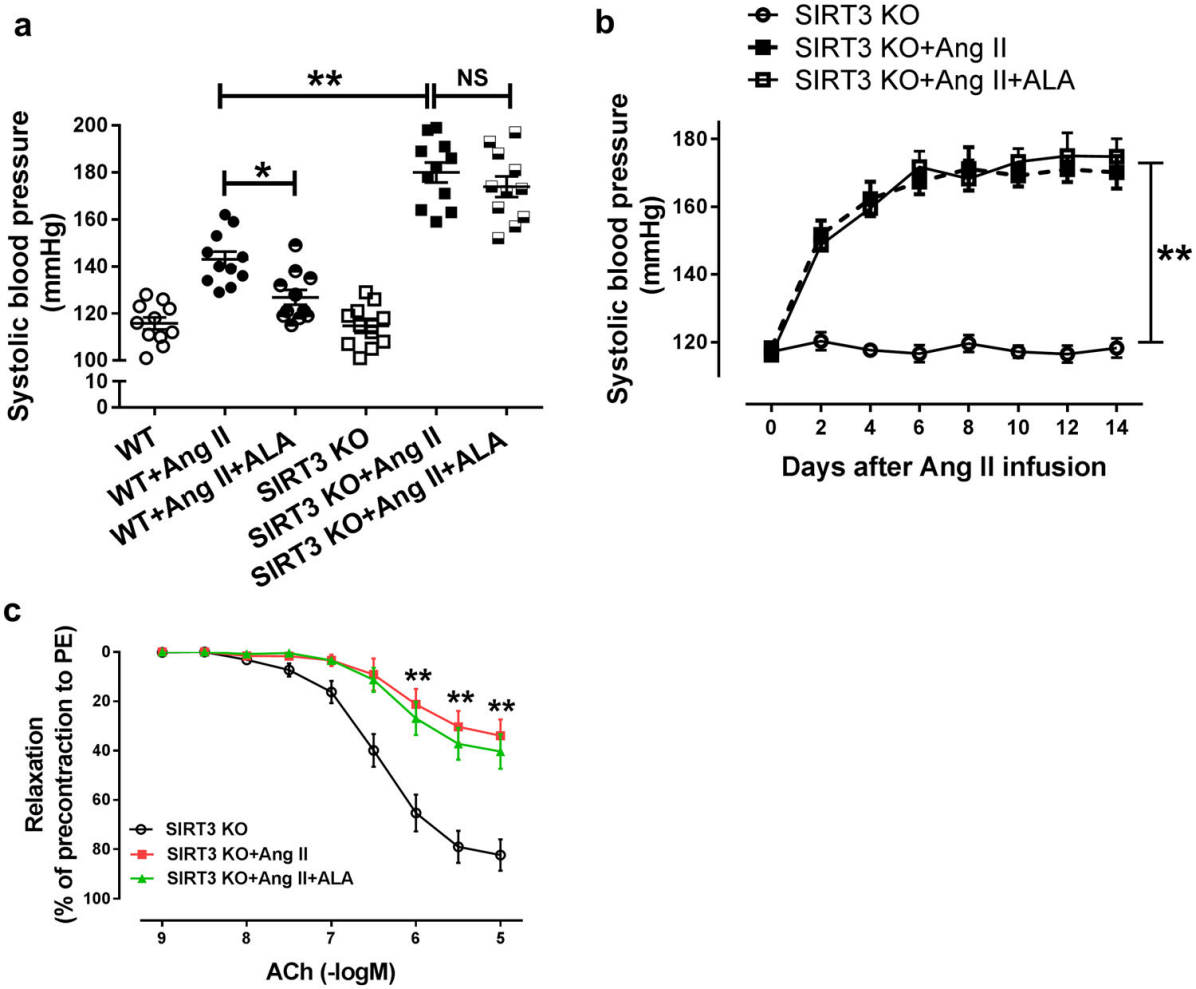
Dietary ALA supplementation exerts anti-hypertensive and endothelium-beneficial effects by rescuing SIRT3 impairment. Eight-week-old SIRT3 KO mice or WT littermates were fed with control or ALA-supplemented diets for 4 weeks, and then randomized to receive AngII (0.4 mg/kg/d) or vehicle infusion for 2 weeks via an osmotic minipump. **a** Systolic blood pressure measurement by direct catheter in SIRT3 KO or WT after 2 weeks of AngII infusion (*n* = 11 animals/group). ^*^P < 0.05 *vs*. WT + Ang II; ^**^P < 0.01 vs. WT + Ang II. **b** Systolic blood pressure was measured by tail-cuff methods during 2 weeks of AngII infusion (*n* = 11 animals/group). **c** Measurement of ACh-induced, endothelium-dependent vasodilation in aortas from SIRT3 KO and WT mice after 2 weeks of AngII infusion (*n* = 9 animals/group). Data are presented as means ± SEM; ^**^P < 0.01 vs. SIRT3 KO.

We next used the SIRT3 KO model to determine whether SIRT3 is the key modulator responsible for the beneficial effects of ALA in hypertensive animals. Systolic blood pressure of SIRT3 KO mice was normal and comparable to WT mice. However, SIRT3 KO mice developed severe hypertension to 180 mmHg in response to low-dose AngII (0.4 mg/kg/d, 2 weeks) infusion, while AngII (0.4 mg/kg/d, 2 weeks) infusion increased SBP of WT mice to 143 mmHg as measured by carotid artery catheterization method (Fig. 5a). Interestingly, ALA supplementation significantly reduced AngII infusion-induced SBP elevation in WT mice, while the antihypertensive effect of ALA supplementation was abolished in SIRT3 KO mice, as measured by both tail-cuff and carotid artery catheterization method (Fig. 5a, b). In addition, AngII infusion significantly impaired the endothelium-dependent vasorelaxation evoked by ACh in aortas from SIRT3 KO mice, while ALA supplementation showed no benefits (Fig. 5c). These data have indicated that SIRT3 plays an important role in the anti-hypertensive and endothelium-beneficial effects of ALA in hypertensive animals.

## Discussion

Our findings suggest that ALA but not LA supplementation rescues SIRT3 reduction, improves endothelial dysfunction and reduces blood pressure elevation in hypertensive animals, thus providing the proof of principle for the benefits of dietary ALA intake against hypertension. SIRT3 plays an important role in the anti-hypertensive effects of ALA by alleviating autophagic flux impairment and inhibiting mitochondrial ROS overproduction in endothelial cells.

Epidemiological studies have revealed an inverse association between dietary intake of long chain n-3 PUFAs and cardiovascular diseases^21^. The three most commonly consumed n-3 PUFAs are ALA, eicosapentaenoic acid (EPA) and docosahexaenoic acid (DHA). Increased consumption of marine-derived EPA and DHA exerts anti-arrhythmic, lipid-lowering, antihypertensive and anti-thrombotic effects^22–24^. However, the availability of EPA and DHA remains restricted in many countries due to unfavorable geography, expensive supply and cultural preference. Therefore, ALA, a plant-derived n-3 fatty acid, may provide an ideal cardioprotective alternative to marine-derived n-3 fatty acid. Given that ALA can be catabolized into EPA and DHA by the body after absorption, it is controversial whether the cardiovascular effects upon ALA dietary intake reflect the activity of ALA or the activity of its longer-chain n-3 fatty acid derivatives. Although biochemical pathways exist to convert ALA to EPA and DHA, such endogenous conversion is limited in humans: between 0.2 and 8% of ALA is converted to EPA and 0 to 4% of ALA to DHA^25–27^. In the PREDIMED study, dietary intake of ALA still conferred protection against cardiovascular disease and all-cause mortality even in a background of high intake of marine-derived long-chain n-3 PUFAs^28^. Our in vitro experiments also indicated that ALA exerted direct antioxidant effect and restored autophagic flux in endothelial cells, which might contribute to the improved endothelial dysfunction and decreased blood pressure in hypertensive animals afforded by ALA supplementation. Therefore, in addition to its derivatives (EPA and DHA)-dependent cardioprotective effects, ALA may exert direct benefits against hypertension.

In contrast to the recognized benefits of n-3 PUFAs, there is still controversy regarding the cardiovascular effects of n-6 PUFAs. Some investigators speculated that a large amount of n-6 PUFA consumption may promote the incidence and development of cardiovascular disease, which may result from its potential pro-inflammatory and thrombogenic properties^7,29^. Observational studies also yielded conflicting results about the relationship between LA intake and blood pressure in humans^30,31^. In the present study, LA supplementation had no effects on blood pressure and endothelial dysfunction in SHRs, which may be partially attributable to the change of prostacyclin from dilator to constrictor in the LA metabolism of SHRs. LA is a precursor of AA, and prostacyclin is the principal metabolite of AA released by ACh in the vessels. Prostacyclin has been reported to be an endothelium-derived vasodilator and is involved in blood pressure regulation^32^. The anti-hypertensive action of LA is partially mediated through prostacyclin metabolism^33,34^. However, prostacyclin cannot evoke vasodilation in aortas from SHRs, and in contrast, it exerts a vasocontracting effect by activating thromboxane A2/endoperoxide thromboxane-prostanoid receptor^35,36^. Different from our studies, an anti-hypertensive effect of LA was observed in the hypertensive animal model of Dahl S and deoxycorticosterone acetate-salt hypertensive rats^37,38^. Noteworthy, a relatively higher LA diet (20% safflower oil) was used in these studies, while 10% safflower oil was used in our study. Another point we should make it clear is that we only included male rats in the present study. Compelling evidence has demonstrated that sex hormones are involved in the regulation of cardiovascular function and play pathological roles in the development of cardiovascular disease^39^. In particular, estrogen at a physiological level was demonstrated to confer protection against cardiovascular diseases including hypertension and ischemia/reperfusion-induced myocardial injury^40,41^. Premenopausal females have been shown both clinically and experimentally to be less sensitive to hypertension compared to the males. In addition, a clear sex difference in many aspects of cardiovascular function in SHRs has been demonstrated previously, including the degree of hypertension and vascular function^42^.

The Sirtuins are a family of NAD^+^-dependent deacetylases and ADP-ribosyltransferases, which have been extensively implicated in modulating a myriad of cellular processes, including energy metabolism, stress responses and cell survival^43^. SIRT3, located in the mitochondria, has been demonstrated to orchestrate global mitochondrial lysine acetylation, enhance antioxidant defense and preserve mitochondrial function^44^. SIRT3 regulates cellular redox homeostasis through deacetylation and activation of SOD2, the mitochondrial isoform of major superoxide-scavenging enzyme. Clinical studies have revealed that SIRT3 expression decreases by 40% by 65 years of age^45^, which is paralleled with the increased incidence of hypertension. SIRT3 deficiency and redox inactivation of SIRT3 result in SOD2 inactivation and contribute to the pathogenesis of endothelial dysfunction and hypertension^10^. In our study, ALA supplementation rescued SIRT3 reduction and SOD2 hyperacetylation, improved endothelial dysfunction and reduced blood pressure elevation in hypertensive animals. SIRT3 knockout rendered the mice more susceptible to AngII-induced hypertension and abolished the endothelium-beneficial and antihypertensive effects of ALA supplementation, indicating the critical role of SIRT3 in vascular protective effects of ALA.

Emerging evidence indicates that basal autophagy is an important in vivo process regulating proper cardiovascular homeostasis and function; either excessive or insufficient levels of autophagic flux can contribute to the pathogenesis of cardiovascular disease^46,47^. Aberrant autophagic activity has recently been implicated in the pathogenesis of hypertension-related endothelial dysfunction^48^. In the present study, the expression of both LC3-II and p62 were upregulated in aortas from SHRs and in endothelial cells exposed to AngII plus TNFα treatment. Given that the autophagosome is an intermediate structure in a dynamic autophagic pathway, the number of autophagosomes observed at any specific time point is a function of the balance between the rate of their generation and the rate of their conversion into autolysosomes^49^. So, increased LC3-II levels may be indicative of either enhanced autophagy induction or defective autophagosomes clearance. In order to identify the exact point of dysregulation of autophagic flux, we further applied the lysosomal enzyme inhibitors E64d and Pepstatin A to impede autophagosome clearance. Interestingly, blockade of autophagosome clearance did not further increase LC3-II levels in endothelial cells exposed to AngII plus TNFα, indicating a defective autophagosome clearance in these cells. The syntropic change of LC3-II and p62 also happened in other pathological models, such as amyloidogenic light chain-mediated cardiotoxicity, ischemia/reperfusion-induced myocardial injury and ethanol-induced myocardial injury, indicating an impaired autophagic activity^50–52^.

Similar to autophagy-related genes, the expression and activity of Sirtuins are influenced by stress and nutrient availability. Sirtuins have been shown to be involved in regulating the autophagy-lysosome pathway^53^. In particular, SIRT1 was demonstrated to regulate autophagy directly through deacetylation of the autophagy-related genes ATG5, ATG7 and ATG8^11^. SIRT1 has also been demonstrated to induce the expression of autophagy pathway components via the activation of FoxO transcription factors^54^. However, less information is available about the association between SIRT3 and endothelial autophagy in hypertension. Our data demonstrate that endothelial SIRT3 reduction is concomitant with dysregulation of autophagic flux in endothelial cells of hypertension, and both could be rescued by ALA administration. More interestingly, silencing SIRT3 blunted the beneficial effect of ALA on autophagic flux in endothelial cells, indicating SIRT3 as an important factor in ALA-regulated autophagy.

In conclusion, our data have demonstrated that ALA supplementation ameliorates endothelial dysfunction and hypertension by rescuing SIRT3 impairment and thus alleviating SOD2 hyperacetylation and autophagic impairment to inhibit vascular oxidative stress, suggesting a novel cardioprotective effect of ALA. These findings could be of interest in translational medicine, where ALA may be applicable in nutritional interventions for the prevention and treatment of hypertension.

## Supplementary information

Supplementary Figure Legends

Supplementary Figure. 1

Supplementary Figure. 2

Supplementary Figure. 3
