# Supplementary material for: α-Linolenic acid but not linolenic acid protects against hypertension: critical role of SIRT3 and autophagic flux
Source: Cell Death Dis. 2020 Feb 3;11(2):83. doi: 10.1038/s41419-020-2277-7 (PMC6997421; doi:10.1038/s41419-020-2277-7)
Supplement: Supplementary file 1 — Supplementary Figure Legends [file 41419_2020_2277_MOESM1_ESM.docx]

**Supplementary Figure Legend**

**Figure S1. Metabolic characteristics of SHRs and age-matched WKYs fed with control, LA-supplemented or ALA-supplemented diets.** a. Body weight of rats in different groups are shown. n = 12 animas per group. b. Serum lipid profiles in different groups are shown. Linoleic acid (LA), arachidonic acid (AA), α-linolenic acid (ALA), eicosapentaenoic acid (EPA) and docosahexaenoic acid (DHA). n-3 fatty acids: ALA, EPA and DHA; n-6 fatty acids: LA, AA. n = 6 animas per group c, d. Fasting blood glucose (c) and fasting serum insuin level (d) are respecitively shown in rats of different groups. n = 12 animas per group. Data are expressed as means ± SE; ^##^ *P*<0.01 *vs*. WKY. ^*^ *P*<0.05, ^**^ *P*<0.01 *vs*. SHR.

**Figure S2. LA supplementation did not significantly influence aortic SIRT3 expression and SOD2 acetylation in SHRs.** a-c. Western-blot analysis of the aortic expression of SIRT3, Ac-SOD2 and SOD2 in SHRs fed with LA-supplemented or control diet (NS: no significance). Data are presented as means ± SEM; n = 6 rats per group.

**Figure S3. Autophagic flux impairment contributes to mitochondrial ROS overproduction in AngII plus TNFα-exposed endothelial cells.** In this section, E64d and Pepstatin A were applied to inhibit lysosomal enzymes and impede autophagosome clearance, and rapamycin was applied to enhance autophagosome clearance to restore autophagic flux. Mitochondria were imaged by MitoTracker Green (100 nM) and mitochondrial ROS production was evaluated by MitoSOX (10 µM, red fluorescence) in endothelial cells. a. Western blot analysis of LC3-II in vehicle- and AngII plus TNFα-treated HAECs with or without lysosomal enzymes inhibition by E64d (5 µg/ml) and Pepstatin A (5 µg/ml). b. Representative immunofluorescent staining of mitochondrial ROS by MitoSOX (10 µM) showing that lysosomal enzymes inhibition by E64d and Pepstatin A promoted mitochondrial ROS production in endothelial cells. Scale bars, 20 µm. Data are presented as means ± SEM from 3 independent experiments. ^**^ *P*<0.01 *vs*. Control. c. Western blot analysis of p62 and LC3-II in AngII plus TNFα-treated HAECs with or without ALA (25 µM) or rapamycin (10 nM) administration. d. Representative immunofluorescent staining of mitochondrial ROS by MitoSOX (10 µM) showing that enhancing autophagosome clearance by rapamycin (10 nM) attenuated mitochondrial ROS overproduction in AngII plus TNFα-treated endothelial cells. Scale bars, 20 µm. Data are expressed as means ± SEM from 3 independent experiments. ^**^ *P*<0.01 *vs*. AngII+TNFα.
