# Supplementary figures and images for: α-Linolenic acid but not linolenic acid protects against hypertension: critical role of SIRT3 and autophagic flux
Source: Cell Death Dis. 2020 Feb 3;11(2):83. doi: 10.1038/s41419-020-2277-7 (PMC6997421; doi:10.1038/s41419-020-2277-7)

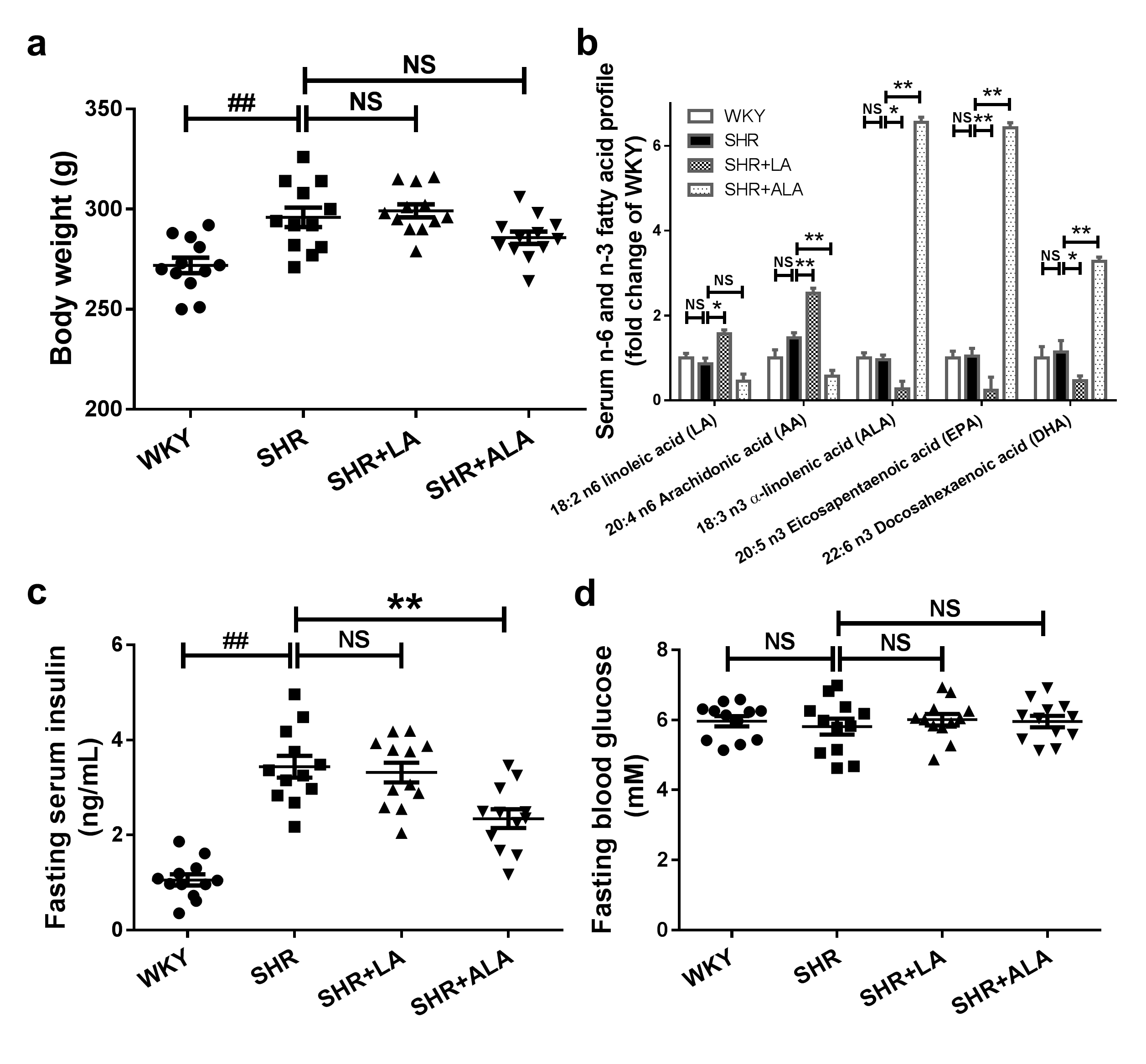

Supplement: Supplementary file 2 — Supplementary Figure. 1 [file 41419_2020_2277_MOESM2_ESM.tif]

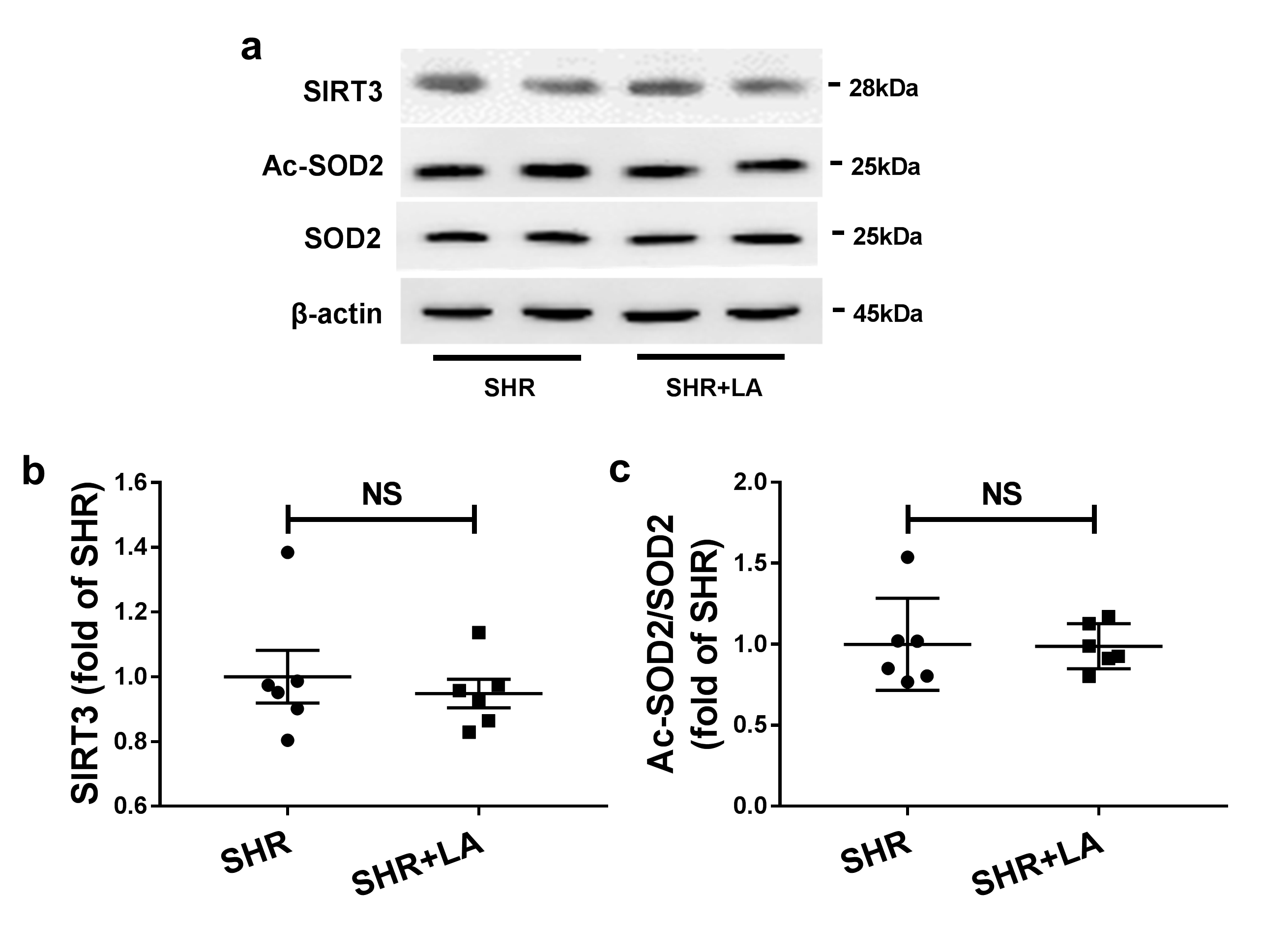

Supplement: Supplementary file 3 — Supplementary Figure. 2 [file 41419_2020_2277_MOESM3_ESM.tif]

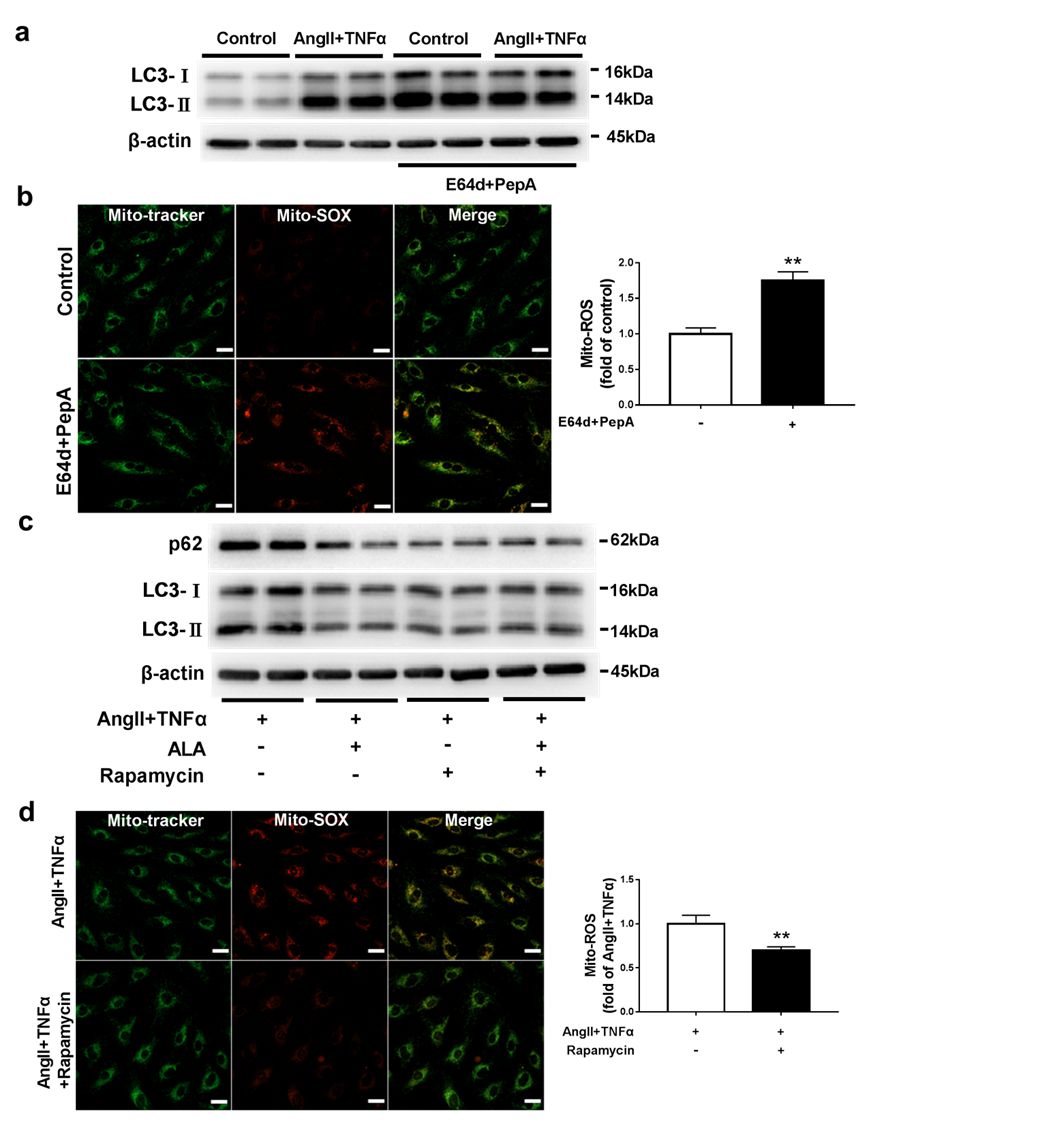

Supplement: Supplementary file 4 — Supplementary Figure. 3 [file 41419_2020_2277_MOESM4_ESM.tif]
